# Dyscalculia, Dysgraphia, and Left-Right Confusion from a Left Posterior Peri-Insular Infarct

**DOI:** 10.1155/2014/823591

**Published:** 2014-04-10

**Authors:** S. Bhattacharyya, X. Cai, J. P. Klein

**Affiliations:** ^1^Department of Neurology, Brigham and Women's Hospital, 45 Francis Street, Boston, MA 02115, USA; ^2^Department of Neurology, Massachusetts General Hospital, Boston, MA 02114, USA; ^3^Harvard Medical School, Boston, MA 02115, USA; ^4^Department of Radiology, Brigham and Women's Hospital, Boston, MA 02115, USA

## Abstract

The Gerstmann syndrome of dyscalculia, dysgraphia, left-right confusion, and finger agnosia is generally attributed to lesions near the angular gyrus of the dominant hemisphere. A 68-year-old right-handed woman presented with sudden difficulty completing a Sudoku grid and was found to have dyscalculia, dysgraphia, and left-right confusion. Magnetic resonance imaging (MRI) showed a focus of abnormal reduced diffusivity in the left posterior insula and temporoparietal operculum consistent with acute infarct. Gerstmann syndrome from an insular or peri-insular lesion has not been described in the literature previously. Pathological and functional imaging studies show connections between left posterior insular region and inferior parietal lobe. We postulate that the insula and operculum lesion disrupted key functional networks resulting in a pseudoparietal presentation.

## 1. Introduction

Since Gerstmann asserted in the early 1900s that a syndrome of finger agnosia, left-right confusion, dysgraphia, and dyscalculia has localizing value, this complex of symptoms has generally been attributed to lesions near the angular gyrus of the dominant hemisphere [1, 2]. The importance of grouping this set of symptoms and the localizing value, however, has been challenged both by clinical experience and through advancements in imaging allowing for more precise anatomic localization of deficits. Of the four originally described components of the syndrome, dyscalculia is encountered most frequently followed by left-right disorientation, dysgraphia, and finger agnosia [2]. In addition to the originally described left parietal focus, the syndrome, often with additional deficits, has also been described in left posterior frontal, temporal, occipital, and thalamic lesions [3]. Here we present a unique case of partial Gerstmann syndrome (dyscalculia, dysgraphia, and mild left-right confusion) originating from a left posterior insular and temporoparietal operculum infarct.

## 2. Case Presentation

**Figure 1:**
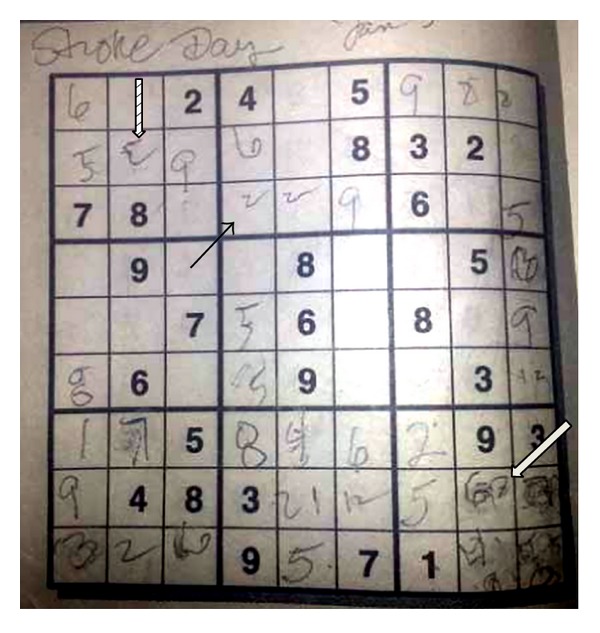
Sudoku grid patient was completing as she had stroke. She played well initially but then could not finish the game. The incomplete puzzle shows scrawled writing (black arrow), inverted digit “3” (arrow with hash marks), and multiple incorrect attempts (white arrow).

A 68-year-old right-handed woman with history of migraine headaches and patent foramen ovale (PFO) on clopidogrel presented with sudden onset of difficulty in completing a Sudoku grid. (Sudoku is a game consisting of a 9 × 9 grid subdivided into nine 3 × 3 subgrids. The objective for each subgrid, row, and column is to contain the digits 1–9 without repetition. The game typically starts with a partially filled grid having a unique solution.) Proficient at baseline, she abruptly found herself unable to fill in the squares (Figure 1). She reported difficulty in calculations and in using her pen. The partially filled Sudoku shows incorrect attempts (Figure 1, open black arrow), coarse writing (Figure 1, black arrow), and the number three written as mirror image (Figure 1, hash mark arrow). Prior to coming to the hospital, she also attempted to shave her legs. She had difficulty using the razor and sustained several superficial lacerations to her left leg.

**Figure 2:**
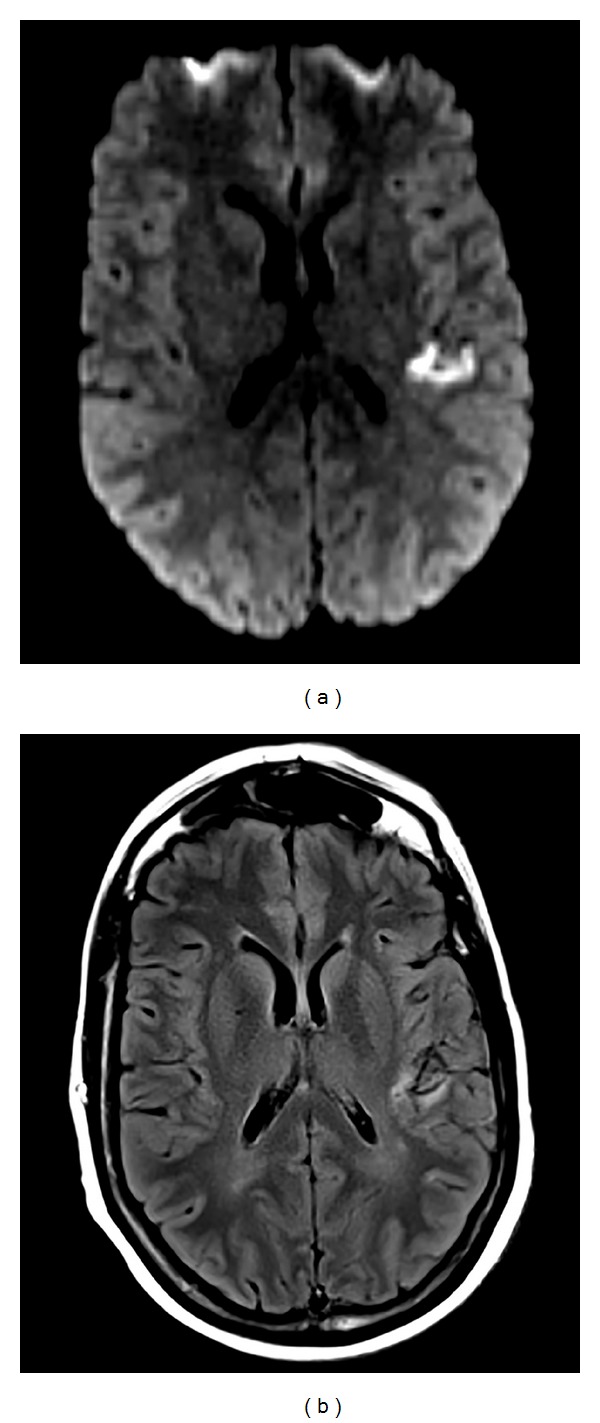
Magnetic resonance imaging (MRI) shows abnormal reduced diffusivity (a) and T2 hyperintensity (b) in the left posterior insula and temporoparietal operculum.

At the hospital, she had good attention and could speak fluently and name, comprehend, repeat, read, and identify her fingers accurately. Her difficulty in writing persisted. For example, intending to write “5,” she instead wrote “15” and she wrote “hong” instead of “long.” She also had difficulty in calculations and could not produce more than one correct answer in a task of serial subtraction by sevens or calculate amount of money in nine quarters. There was one error in three left/right discrimination tasks (incorrectly stated left for right). She also initially reported being left-handed instead of right-handed. There was otherwise no visual or sensory neglect of the left side, and there were no acute changes in muscle strength, skin sensation, or coordination in her limbs. Magnetic resonance imaging (MRI) performed eleven hours after onset of symptoms showed a focus of abnormal reduced diffusivity within the left posterior insula and temporoparietal operculum consistent with acute ischemic infarction (Figure 2).

Over the next two days, her ability to calculate improved although she continued to have difficulty in serial subtraction tasks and accurate completion of Sudoku grids. In poststroke follow-up after three weeks, she reported twice daily sensation of a rising sense of heat in her body followed by period of confusion and sense of doom. Electroencephalography (EEG) showed bitemporal slowing worse on the left side. Topiramate was initiated for presumed focal onset seizures, and the periodic sensation of rising warmth abated though difficulty with calculations persisted. Stroke workup including 24-hour continuous electrocardiographic monitoring, transthoracic echocardiography, and cervical and intracranial MR angiography revealed only a known patent foramen ovale. Subsequent imaging of the lower extremity and pelvic veins showed no thrombosis, but May-Thurner anatomy was noted. For presumed paradoxical thromboembolism, she was started on anticoagulation five days after her stroke with plan to continue indefinitely.

## 3. Discussion

Insular and peri-insular infarctions are known to produce a wide range of motor disturbances, somatosensory syndromes, and language deficits. In a meta-analysis of clinical symptoms associated with peri-insular strokes from institutional case series and published cases (23 patients), the predominant symptoms were dysarthria (10 patients, 43%), somatosensory deficits (10 patients, 43%), and aphasia (10 patients, 43%) [4]. Sensory deficits were primarily numbness and dysesthesia while aphasia consisted mostly of anomia and phonemic paraphasias. Other less common manifestations included a vestibular-like syndrome (8 patients, 35%), contralateral weakness (6 patients, 26%), dysautonomia (4 patients, 17%), and gustatory disturbances (3 patients, 13%) [4]. The wide variation of symptoms in this series and others is explained partially by whether the lesion affects the anterior or posterior insula and by the laterality of the lesion. Infarction of the posterior insular cortex has been linked to contralateral pseudothalamic sensory syndrome as well as pseudovestibular vertigo and fluent aphasia [4, 5].

Our patient, however, presented with profound dyscalculia accompanied by milder dysgraphia and left-right confusion from a left posterior insular and temporoparietal operculum ischemic infarction. None of these deficits have been traditionally attributed to insular or peri-insular infarcts. The ability to perform calculations—the patient's most impaired faculty—has been studied in both pathological and physiological contexts. Lesion studies in patients with acquired dyscalculia have primarily implicated the left parietal cortex, particularly near the angular gyrus [6, 7]. Functional studies of calculation show a more complex activation pattern. In a task requiring subtraction by sevens, functional MRI (fMRI) showed preferential activation of left dorsolateral prefrontal cortex and bilateral inferior parietal cortices when controlled for number production [8]. In a previous fMRI study with less robust control of number production versus calculation, bilateral activation in the parietal, prefrontal, and premotor cortices was seen [9]. Studies of developmental dyscalculia using magnetic resonance spectroscopy show signal abnormalities in the left temporoparietal region near the angular gyrus [10].

Additionally, dyscalculia often occurs in the context of Gerstmann syndrome [1]. Our patient had elements of Gerstmann syndrome, including dyscalculia, dysgraphia, and mild left-right confusion (one error in three tasks). She did not have finger agnosia. The agraphia in Gerstmann syndrome can take the form of aphasic agraphia with errors in content of writing, apraxic agraphia manifested as a scrawl, or spatial agraphia seen as errors in management of positioning of letters on paper [2, 3]. Our patient had aspects of apraxia (poorly formed letters) as well as aphasia (letter substitutions) in her writing difficulty. As far as we know, Gerstmann syndrome has not been previously described with insular or peri-insular lesions.

Our patient consequently had a pseudoparietal lobe presentation from a posterior insular and temporoparietal opercular infarct. Based on animal and human pathological studies, the insula is known to have extensive connections both to the cortex and deep structures [11]. With the frontal lobe, the insular cortex has reciprocal connections with frontal operculum, orbitofrontal cortex, and prefrontal cortex and has efferent connections with frontal cortex adjoining the motor areas, inferior frontal gyrus, ventral granular cortex, and Brodmann areas 6 and 12 [12]. With the parietal lobe, the insula has reciprocal connections with the anterior inferior parietal cortex, parietal operculum, primary and secondary somatosensory cortex, and parietal retroinsular region [11]. In a fMRI study of resting insular-parietal network in control subjects, the left posterior insular cortex activity correlated positively with bilateral inferior parietal cortices [13]. The anticorrelated network of the left posterior insular cortex included bilateral precuneus regions of the parietal lobe. The right posterior insular cortex correlated with the adjacent parietal operculum but did not correlate positively or anticorrelate with other additional regions in the parietal lobe [13].

Aside from the frontal and parietal connections described above, the insula also has rich connections with the temporal lobe, cingulate cortex, limbic structures, and multiple thalamic nuclei [11]. To explain our observed pseudoparietal syndrome, we propose that the infarct disrupted the connectivity between the left posterior insular cortex and bilateral inferior parietal lobe regions. With the acute loss of modulating influences from the insular lobe, the patient presented with parietal lobe dysfunction similar to what would be expected from a lesion in the inferior parietal lobe. Since the insular cortex is also connected with multiple other regions including the frontal and temporal lobes, the mechanism of parietal lobe dysfunction may alternatively involve more indirect mechanisms of disrupting frontoparietal or temporoparietal circuitry. An alternative explanation is that the ischemic penumbra of the patient's infarct included the left inferior parietal cortex. While such an explanation could account for her initial symptoms, we would not expect her to have long-term persistent deficits with an infarct restricted to the left peri-insular region.

This patient's poststroke course was complicated by presumed insular or peri-insular seizures manifesting as autonomic sensations of warmth followed by feelings of doom and confusion. The initial autonomic signs are consistent with a peri-insular epileptic focus though similar ictal sensations can be caused by lesions in the amygdala, anterior cingulum, and supplementary sensorimotor areas [14]. Her autonomic sensation was followed by the psychic sensation of fear, which has been linked during brain stimulation studies to activation of the amygdala, hippocampus, temporal cortex, and mesial temporal region [15]. In her case, the fearful sensation probably represented spread of the epileptic focus from the insular region to other temporal lobe structures. While her EEG did not show overt seizures or abnormal electrographic discharges, epileptic foci in the insular region can often manifest solely as slowing on surface EEG [16]. The dyscalculia was not likely an ictal phenomenon since it persisted despite antiepileptic drug therapy and cessation of the spells of confusion.

This unique presentation of pseudoparietal syndrome and “dys-sudokia” resulting from a left posterior peri-insular infarct raises a few final points. The patient presented to the hospital about eleven hours after the onset of symptoms. She already had abnormal T2 hyperintensity in the region of the infarct suggesting that the infarct had been present for at least several hours. She may have had additional symptoms that were not noticed or reported. Furthermore, her MRI also had scattered abnormal T2 hyperintensities throughout the subcortical and periventricular white matter, without reduced diffusivity. These abnormalities could represent prior subclinical pathological changes which influenced the presentation of this infarct. Finally, without functional imaging data we are not certain of her hemispheric dominance with respect to language. Even in extreme right-handed healthy subjects, right brain language dominance is present in about 4% when increased blood flow is used as a surrogate for brain activity [17]. Altered dominance or even codominance could have influenced her initial symptomatology.
